# Sex‐dependent APOE4 neutrophil‐microglia interactions drive cognitive impairment in Alzheimer's Disease

**DOI:** 10.1002/alz.087776

**Published:** 2025-01-03

**Authors:** Oleg Butovsky

**Affiliations:** ^1^ Brigham and Women’s Hospital, Harvard Medical School, Boston, MA USA

## Abstract

**Background:**

One of the major outstanding questions in the field of Alzheimer’s disease (AD) research is the underlying mechanism by which APOE ε4, the strongest genetic risk factor for AD, contributes to disease pathogenesis. Current therapies targeting amyloid‐beta plaques show modest effect in non‐APOE4 male AD patients, and greatly increase the risk for amyloid‐related imaging abnormalities – edema/effusion (ARIA‐E) in APOE ε4 carriers. We made an important discovery that APOE4 neutrophil‐microglia interactions drive cognitive impairment in a sex‐dependent manner.

**Method:**

Microglia isolated from AD brains and blood neutrophil transcriptomics of 142 patients across APOE variants in both sexes, utilizing snRNASeq and multiplex flow cytometry and validation in two independent cohorts of APOE4 female AD patients. Reanalysis of an independent cohort of 422 human blood samples, implementing single cell deconvolution tools. Utilizing humanized AD mouse models, we extended these findings and addressed the mechanism by which APOE4 neutrophils suppress brain microglia.

**Result:**

We identified neutrophils degranulation as the most affected pathways in microglia isolated from AD brains of APOE ε4 females and a new subset of neutrophils associated with cognitive impairment in APOE4 patients. This phenotype is defined by increased IL‐17 and IL‐1 co‐expressed gene modules in blood neutrophils of cognitively impaired female APOE ε4 carriers, showing increased infiltration to the AD brain. APOE4 female IL‐17^+^ neutrophils upregulated the immunosuppressive cytokines IL‐10 and TGFβ, and immune checkpoints including LAG‐3 and PD‐1, associated with accelerated immune aging. Deletion of APOE4 in neutrophils reduced this immunosuppressive phenotype and restored microglial response to neurodegeneration (MGnD), limiting plaque pathology in AD mice. Mechanistically, we show that IL‐17F upregulated in APOE4 neutrophils interacts with microglial IL‐17RA to suppress the induction of MGnD phenotype, and that blocking this axis supported cognitive improvement in AD mice.

**Conclusion:**

These findings highlight the impact of peripheral immunity on AD pathology, provide a translational basis for precise targets for APOE ε4 carriers, and identify the biological rational for IL‐17F blockade in females with cognitive impairment. These findings link neutrophil signature to sex and APOE4 as drivers of AD and emphasize the necessity for personalized therapeutic interventions for AD.

